# Predictive Brain Activity Shows Congruent Semantic Specificity in Language Comprehension and Production

**DOI:** 10.1523/JNEUROSCI.1723-23.2023

**Published:** 2024-01-24

**Authors:** Luigi Grisoni, Isabella P. Boux, Friedemann Pulvermüller

**Affiliations:** ^1^Brain Language Laboratory, Department of Philosophy and Humanities, Freie Universität Berlin, Berlin 14195, Germany; ^2^Cluster of Excellence ‘Matters of Activity, Image Space Material’, Humboldt Universität zu Berlin, Berlin 10099, Germany; ^3^Berlin School of Mind and Brain, Humboldt Universität zu Berlin, Berlin 10099, Germany; ^4^Einstein Center for Neurosciences, Berlin 10117, Germany; ^5^Biological and Social Psychology, Institute of Psychology, RWTH Aachen University, Aachen 52056, Germany

**Keywords:** language comprehension, language production, prediction potential (PP), predictive processing, semantic processing, EEG

## Abstract

Sentence fragments strongly predicting a specific subsequent meaningful word elicit larger preword slow waves, prediction potentials (PPs), than unpredictive contexts. To test the current predictive processing models, 128-channel EEG data were collected from both sexes to examine whether (1) different semantic PPs are elicited in language comprehension and production and (2) whether these PPs originate from the same specific “prediction area(s)” or rather from widely distributed category-specific neuronal circuits reflecting the meaning of the predicted item. Slow waves larger after predictable than unpredictable contexts were present both before subjects heard the sentence-final word in the comprehension experiment and before they pronounced the sentence-final word in the production experiment. Crucially, cortical sources underlying the semantic PP were distributed across several cortical areas and differed between the semantic categories of the expected words. In both production and comprehension, the anticipation of animal words was reflected by sources in posterior visual areas, whereas predictable tool words were preceded by sources in the frontocentral sensorimotor cortex. For both modalities, PP size increased with higher cloze probability, thus further confirming that it reflects semantic prediction, and with shorter latencies with which participants completed sentence fragments. These results sit well with theories viewing distributed semantic category-specific circuits as the mechanistic basis of semantic prediction in the two modalities.

## Significance Statement

We report larger anticipatory negative-going prediction potentials (PPs) after sentence fragments with predictable than unpredictable endings both during language comprehension and production. In production and comprehension experiments, PP topographies resembled each other, but, for each modality, PPs differed in the same way between semantic categories of the predictable words. Likewise, cortical source estimation revealed similar prediction-related cortical activations across modalities but consistent activation differences reflecting the meaning of predicted symbols. Furthermore, PP size was linked with behavioral measures of predictability (cloze probability) and ease of processing (reaction times), and correlated PPs were seen in production and comprehension; these observations are consistent with similar prediction mechanisms across modalities but different ones for semantic types.

## Introduction

Perception arises from information reaching the primary sensory cortex cascading “upward” to higher perceptual and multimodal areas (Hubel, 1995; Riesenhuber and Poggio, 2002; DiCarlo et al., 2012). However, recent research emphasizes the importance of predictive processing, which influences sensory activation in a “top-down” manner and leads to priming-like effects in case of matches, but prediction error signals in case of mismatching between predictions and actual input (Knill and Pouget, 2004; Friston, 2010; Clark, 2013; Grisoni et al., 2016; Auksztulewicz et al., 2017; De Lange et al., 2018; Keller and Mrsic-Flogel, 2018). As separable mechanisms of prediction and prediction error are fundamental to predictive processing theories, assessing these models against bottom-up approaches requires separable measures of predictive brain activity and prediction error signals (Walsh et al., 2020). In the spatial domain, it is difficult to unambiguously identify and separate “prediction units” from “prediction error units” (Walsh et al., 2020), as recent invasive neurophysiological studies seemingly supporting this distinction (Bell et al., 2016, 2017) raised discussion about alternative interpretations (Vinken and Vogels, 2017; Kaposvari et al., 2018; Walsh et al., 2020). However, a straightforward separation is possible in the time domain. Predictive activity *precedes* predictable stimuli, whereas prediction error signals can only emerge *after* the critical stimulus arrives. A range of recent noninvasive neurophysiological studies revealed separate and distinct brain indexes of prediction, the so-called PPs, which precede predictable (but not unpredictable) stimuli (Peelen and Kastner, 2011; Kok et al., 2014, 2016; Grisoni et al., 2016, 2017, 2021; Trapp et al., 2016; León-Cabrera et al., 2017, 2019, 2021) and even reflect predicted information at different levels, such as the acoustic–phonological makeup of spoken words (Grisoni and Pulvermüller, 2022), semantic meaning (Grisoni et al., 2021), and communicative function (Boux et al., 2021). These prediction-related measures show regular relationships to established brain responses recently linked to prediction error, such as the mismatch negativity (Näätänen et al., 2007; Bendixen et al., 2012; Grisoni et al., 2016, 2019a) and the N400 (Kutas and Federmeier, 2011; Lau et al., 2013; Grisoni et al., 2017, 2021).

Predictions are effective in perception and production. In production, the planning of a voluntary movement includes predictions about coordinated muscle contractions, reafferent sensory signals, and goal-related aspects of the action. Relevant models can be partitioned into three groups: (1) classic cascaded bottom-up perception and top-down production models see different mechanisms at work in production and perception (e.g., in the language domain, see Levelt, 1993; Indefrey and Levelt, 2004; Hickok and Poeppel, 2007; Indefrey, 2011; Price, 2012; Hickok, 2013), thus implicating different anticipatory mechanisms; (2) major predictive processing accounts propose the same prediction mechanisms in perception and production, which are localized in one (set of) areas (e.g., prediction units in the sensorimotor system, Schubotz, 2007; Pickering and Clark, 2014); and finally (3) predictions may be so fundamental that they can involve all brain areas (De Lange et al., 2018; Pulvermüller and Grisoni, 2020; Grisoni, 2022); in this perspective, prediction mechanisms are realized as preactivity in neuron circuits whose distribution across the cortex varies dependent of which type of information the circuit “holds.” For example, different cortical activation topographies were observed for meaningful words predictable from their preceding context, depending on whether these words designate animals and therefore denote substantial amounts of visual information (about shape and color) or tools, therefore implicating significant additional action knowledge (for discussion, see Pulvermüller, 1999; Kiefer and Pulvermüller, 2012; Grisoni et al., 2021). Interestingly, these prediction-related semantic activation differences resemble those revealed by brain responses to single words presented out of context (Martin et al., 1996; Kiefer, 2005; Martin, 2007; Binder and Desai, 2011; Moseley and Pulvermüller, 2014; Pulvermüller, 2018; Kuhnke et al., 2020; Grisoni et al., 2021). This congruency of category-specific semantic activations in prediction and recognition is consistent with meaning-specific prediction mechanisms, rooted in the activation of topographically specific cortical circuits processing these meaningful signs.

We here ask whether statistically different prediction signals index the planning of a specific action and the expectation of a specific perception—the production or understanding of a context-determined word—and whether predicted words with different meanings, animal and tool words, elicit consistently different predictive semantic brain activity in speaking and understanding. The classic position [different production and perception mechanisms, see (1) above] implies different activity patterns for prediction in production and perception, but not necessarily between different semantic word types. A single-locus prediction model [e.g., prediction in the sensorimotor cortex, see (2)] suggests no differences between modalities or between semantic types. Finally, models postulating prediction mechanisms in distributed neuronal circuits with different cortical topographies [see (3) above] do not predict differences between prediction mechanisms in production and perception but distinct brain indexes for different semantic types. We used 128-channel EEG to record event-related anticipatory PPs and calculated distributed cortical sources to evaluate these predictions.

## Materials and Methods

### Participants

Thirty healthy adults (mean age, 24.7 years; range, 18–35 years; 15 females) participated in the study. All subjects were German native speakers with no records of neurological and/or psychiatric disease. Datasets from four subjects were excluded either because of technical problems during EEG recording or noisy data (e.g., >30% of rejected trials) or because the participant did not pronounce any word in too many trials. Therefore, the final sample consisted of 26 participants (mean age, 24.7 years; range, 18–35 years; 13 females) all right-handed (mean laterality quotient 88.2, ±14.6 SD) as documented by the Edinburgh Handedness Inventory (Oldfield, 1971). The experimental procedures were approved by the Ethics Committee of Charité Universitätsmedizin, Campus Benjamin Franklin, Berlin, Germany. All tested participants provided written informed consent and were paid 10 euros per hour for their participation.

### Experimental design

**Figure 1.**
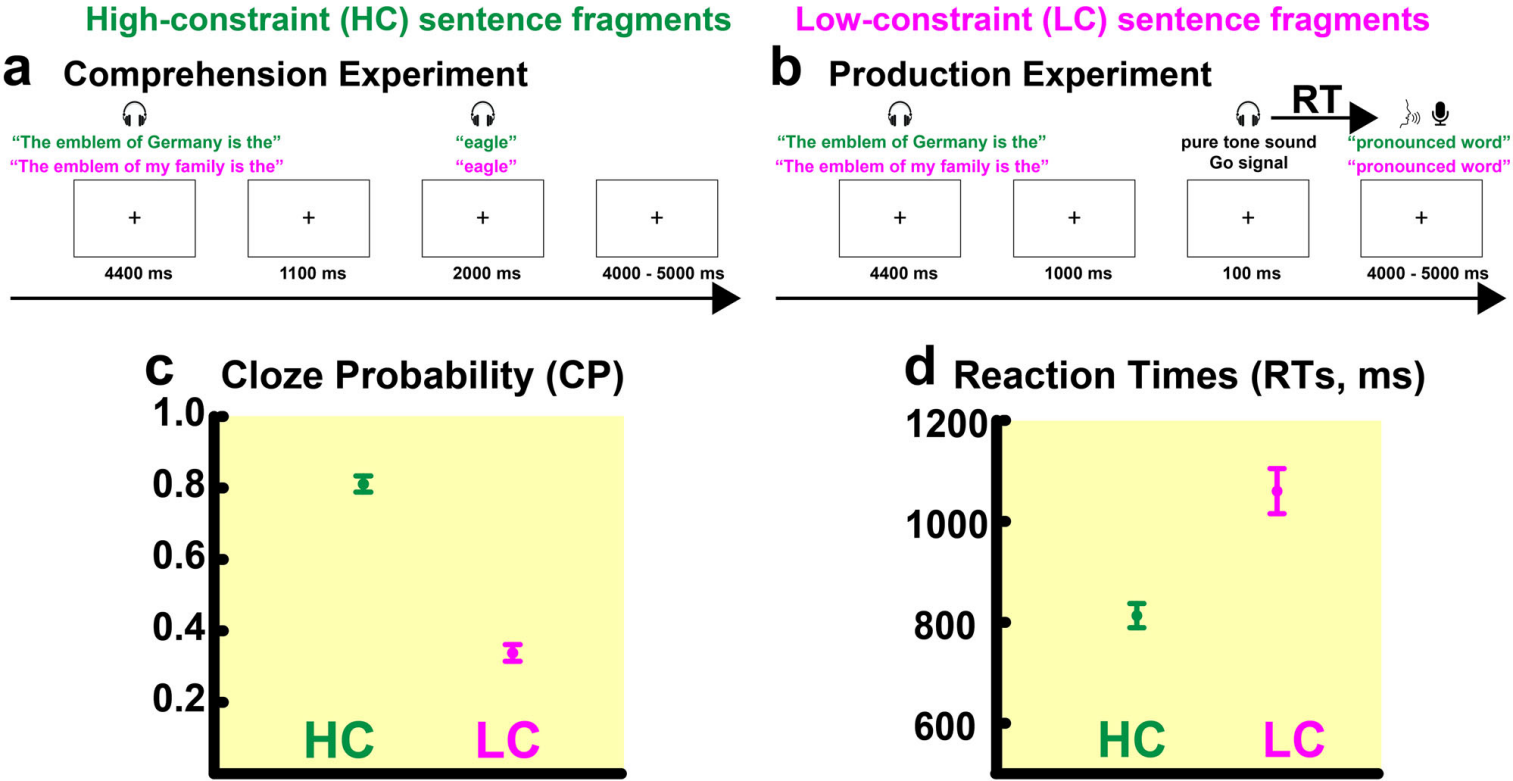
Experimental design and behavioral results. ***a***, Schematic representation of the comprehension experiment in which participants had to passively listen to all the spoken sentences randomly presented. Sentence fragments and the critical target words were separated by a 1.1 s break. ***b***, Schematic representation of the production experiment in which participants heard the sentence fragments and, after a delay of 1 s, heard a “go” signal and then pronounced a word to complete the sentence. ***c***, Behavioral results: CPs for the target words completing the predictable “HC,” in green, and the unpredictable ending “LC,” in magenta, sentences (averages and standard errors of mean). ***d***, Behavioural results: delays between “go” signal onset and the onset of word production in the HC (in green) and LC (in magenta) conditions of the speech production experiment.

Participants were presented with the same 116 German sentences previously used for another work (Grisoni et al., 2021). Half of these sentences (i.e., 58) started with a sentence fragment that strongly predicted the final target word [*high-constraint* (HC) sentences, Fig. 1; example translated from German: “The emblem of Germany is the eagle”], whereas the other 58 sentence fragments allowed several different final target words [*low-constraint* (LC) sentences, Fig. 1; example translated from German: “The emblem of my family is the eagle”]. That the sentences in the two lists each had a predictable versus unpredictable ending was confirmed by a rating study with subjects not partaking in the neurophysiological study (Grisoni et al., 2021). Each of the two lists (HC and LC) included 29 sentences ending in an animal word, such as “eagle,” and another 29 ending in a tool word, such as “horn.” Except for predictability, HC and LC sentences were matched for several important linguistic features, including sentence length, syntactic structure, and verb conjugation, whereas animal and tool, target, words always appeared at the end of the sentences, and they were matched for several linguistic features, such as word length and word frequency (for stimulus evaluation and characteristics, see Grisoni et al., 2021).

The EEG recording took place in the acoustically and electrically shielded chamber of the Brain Language Laboratory at the Freie Universität Berlin. The EEG task was programmed using E-Prime 2.0.8.90 software (Psychology Software Tools). All the acoustic stimuli were presented binaurally, though high-quality headphones (Ultrasone HFI-450 S-LOGIC), at a comfortable hearing level (adjusted per each participant before starting the experiment). During the EEG recording, participants were monitored via a camera.

The study included two experiments, each conducted in one block (Fig. 1). During the comprehension experiment, participants were presented with all the sentences, and they were instructed to listen to them carefully. To separate stimulus-elicited activity to the last words of the sentence fragments from any anticipatory, predictive slow-wave activity, the final target word was presented after a delay of 1,100 ms from sentence fragment offset (Grisoni et al., 2021). Previous studies show that predictive brain activity during language understanding is similarly present when such a pause is introduced before the critical word (León-Cabrera et al., 2019). A further reason to introduce this delay was to match the comprehension experiment with speech production (described below). It had previously been reported (Staub et al., 2015) that, when being presented with sentence fragments in a production task, participants take at least ∼1,000 ms to find the final, critical, word to complete a fragment. To match this break, a pause was added in the comprehension trials. No overt behavioral response was required or recorded during the comprehension EEG experiment. After the comprehension experiment, to ascertain that the participants had paid attention to the sentences, they were asked to indicate which sentences, from a list of 20, they had listened to before. Of the 20 sentences on the list, only 10 had been presented during the EEG recording of the comprehension experiment; EEG participants correctly identified an average of 9.6 sentences (range 8–10). During the production experiment, participants were presented with the same spoken sentence fragments, missing the final target word, which, in this case, was replaced by a sine pure tone (F0, 1,000 Hz) lasting for 100 ms. Participants were instructed to wait until the beep was presented and then to pronounce one single word, which they would use to complete the preceding sentence fragment. The order of the two blocks (i.e., comprehension and production) was counterbalanced across participants so that half of the participants started with comprehension and the other half with production. The sentence order was randomized in three different lists, and each participant was randomly assigned to one of these lists that remained the same across the two blocks (i.e., the same sentences were presented to all participants, only the order varied according to the specific randomization). Similar to the comprehension experiment, the speech production experiment included a delay of 1,000 ms (Fig. 1) between the offset of the sentence fragment and the onset of the beep to reduce as much as possible any overlap of the brain responses elicited by the preceding sentence fragments and by any anticipatory slow-wave activity. The beep presentation functioned as a go signal for participants to select and pronounce a word to complete the incomplete sentences. In both blocks, the interval between individual trials (i.e., sentences) varied randomly between 4,000 and 5,000 ms. The words pronounced during the production experiment were recorded through a microphone placed inside the chamber. Using Audacity 2.1.1 (https://www.audacityteam.org/), three research assistants independently determined off-line the time of speech onset by listening to, and visually inspecting, the waveforms of the audio files recorded during the production experiment. These research assistants calculated, per each participant (i.e., *N* = 30) and per each trial (i.e., *N* = 116), the time interval (i.e., Δ*t*) between the start of the beep and the start of the spoken word. Trials for which the three research assistants indicated latencies that differed by >20 ms (∼10% of the sample) were re-evaluated by the lead investigator (LG). This evaluation had different purposes: (1) to mark and remove production trials during which participants did not utter any words. All these trials, together with their counterparts of the other constraint condition, were excluded from the final analyses of both the production and comprehension experiments to avoid differences in the number of trials between HC and LC conditions and between the two modalities (ANOVA analyses, see below). Overall, ∼17% of all (i.e., 116) production trials were rejected based on this criterion; (2) to create trial-specific triggers at speech onset [see below, Electrophysiological recordings and preprocessing; please note that methods normally used to extract speech onset automatically (including voice keys) did not produce precise enough results; therefore, the more labor-intensive processing was chosen]; (3) to identify the pronounced words to ascertain their status as HC versus LC endings (Grisoni et al., 2021). HC sentence endings were more predictable than those produced in the LC contexts, as confirmed by the statistical evaluation (see Results); (4) to extract the latencies of the target word production, which were later used to determine the processing load or ease of completing the fragments (i.e., response times; Fig. 1).

#### Electrophysiological recordings and preprocessing

The EEG was recorded through 128 active electrodes (actiCAP system, Brain Products). As compared with the standard 10-10 system configuration of the actiCAP, the reference was moved from the FCz position to the tip of the nose (which allows a greater reduction of the impedance), and the electrode occupying the I2 position was moved to the empty FCz position. Furthermore, the vertical electrooculogram (VEOG) channel was obtained by moving the electrode occupying the I1 position to below the right eye, whereas the horizontal electrooculogram (HEOG) signals were recorded with two electrodes embedded in a fabric cap and placed next to the eyes (i.e., F9 and F10). Finally, two electrodes were used as electromyogram (EMG) channels to keep track of articulatory muscle activity during the entire EEG recording. To this end, the electrode OI1 h was removed from its standard position, and it was placed in proximity of the left buccinator muscle (i.e., next to the lateral opening of the mouth), whereas the channel OI2 h was placed in proximity of the left zygomaticus major muscle (i.e., right below the cheekbone). During data acquisition, the electrooculogram (EOG) and EMG channels had the same reference as all the other electrodes.

EEG independent component analysis (ICA)–based correction for eye and articulatory motor artifacts was performed on the original data. The ICA was performed with the default infomax algorithm “runica” (Bell and Sejnowski, 1995), as implemented in EEGLAB 13 (Swartz Center for Computational Neuroscience, http://www.sccn.ucsd.edu/eeglab). Since the (ICA) decomposition of the EEG signal works better for signals filtered at lower frequencies (e.g., <2 Hz; Winkler et al., 2015; Klug and Gramann, 2020), for the ICA, we created two datasets per participant: one dataset contained the EEG signal to which a 0.1 Hz high-pass and an 80 Hz low-pass filters were applied (the target data set), whereas the second dataset (used for ICA purposes only) contained the same EEG data high-pass filtered below 2 Hz (to avoid edge artifacts, the Butterworth zero-phase filter, 24 dB/oct, was always applied to the raw, unsegmented, data). Afterward, raw data were segmented time-locked to the critical events (i.e., the final target word in comprehension, the “go” beep sound in production); for the comprehension experiment, the epochs started at 1,100 ms before to 1,900 ms after the critical event, whereas for the production experiments, the epochs started at 1,100 ms before to 3,900 ms after the beep sound. The decomposition of the signal into the maximally independent components was carried out on the segmented EEG data filtered below 2 Hz, to then associate the obtained ICA arrays with the first target dataset, which is that one filtered below 0.1 Hz. Following this procedure, it is possible to obtain a more accurate IC decomposition without losing the signal at lower frequencies, which might be particularly important for anticipatory slow-wave potentials (Kappenman and Luck, 2012). Therefore, the ICA was carried out on all 128 electrodes to correct the signal for eye movements and articulatory motor activity (Delorme and Makeig, 2004). Artifactual ICs were identified for these two different datasets, that is, (1) comprehension, time-locked to word presentation, and (2) production, time-locked to the beep sound onset. The EEG signals from the comprehension experiment (1) were also corrected for potential articulatory artifactual activity. Indeed, it has been proposed that prediction in language comprehension might originate from the production system (Pickering and Garrod, 2007), and, following this reasoning, it might be possible that the passive listening to sentences induces some anticipatory motor system activation and resultant articulatory artifacts. Therefore, to exclude the possibility that the previously reported prediction potential (PP) responses were due to this artifactual activity, here we also corrected the EEG signals from the comprehension experiment. To this end, ICs were marked as artifactual with a semiautomatic procedure using the algorithm “ICLabel,” which classifies the ICs based on their functional origin (Pion-Tonachini et al., 2019). An IC was marked as artifactual if it was classified either as “eye,” “muscle,” “hearth,” or “other” with a probability >80% by the “ICLabel” algorithm included in EEGLAB (see above). Furthermore, any other component whose topography showed peak activity over HEOG, VEOG, or EMG channels and whose power spectrum smoothly decreased was marked as artifactual by visual inspection (Delorme and Makeig, 2004). All the artifactual (eye and articulatory motor) ICs were subtracted from the EEG signals. On average, 9.7 (range, 1–27) out of 128 components were removed from the comprehension experiment; 40.9 (range, 19–67) out of 128 components were removed from the production experiment. Finally, since the auditory evoked potentials (AEPs) elicited by the “go” signal (beep) overlapped with the slow anticipatory potential shift before the speech onset of the target word in the production experiment, we created a second dataset for the production experiment from which the beep-related auditory evoked potential (AEP) ICs were removed. To this end, the AEP-related ICs were identified and removed according to the following criteria: (1) the ICs activity showed three successive peaks (i.e., P50-N100-P200), and (2) these three peaks emerged at early latencies (i.e., ∼50–100–200 ms). This method follows a recent study by Ross et al., (2022). On average, an additional 2.1 ICs (range, 1–4) of the remaining ICs were removed to create the second dataset of the production experiment.

After signal correction, off-line analysis continued with BrainVision Analyzer 2.2 (Brain Products). After ICA, bipolar EOG channels were created by subtracting the Fp2 from the lower eye electrode, whereas the new HEOG channel was obtained by subtracting the right, F10, from the left, F9, channel. The EEG signal was then epoch time-locked to the event. The EMG channel used for the statistical analysis was computed as the average of the two EMG channels (see above). The epochs were filtered with a low-pass (20 Hz, Butterworth zero-phase filter, 24 dB/oct) and notch filter (50 Hz, 24 dB/oct); the filter settings adopted in this study (i.e., high-pass 0.1 Hz, low-pass 20 Hz, and notch filter 50 Hz) are standard in research on slow brain potentials (Kappenman and Luck, 2012). For the comprehension experiment, epochs lasted from 1,100 ms before to 1,900 ms after target word onset); in the production experiment, epochs lasted from 1,100 ms before until 1,900 ms after beep onset and from 1,100 ms before until 900 ms after speech onset. As a baseline, we always used the first 100 ms of the epoch (i.e., from −1,100 ms to −1,000 ms before the critical event). Epochs with voltage fluctuation exceeding either 100 µV at any channel or 60 µV at EOG channels and those contaminated with artifacts due to amplifier clipping, burst of electromyographic activity, or alpha power were excluded from averaging. On average, ∼5% of trials were rejected.

### Statistical analysis

#### Electrophysiological signals

For all time windows analyzed in this study, we also tested whether the activity recorded at the EMG channels (average of the two EMG channels, see above), at the same latencies, differed between the two conditions (i.e., HC and LC).

#### Comprehension and production experiments

##### Anticipatory brain activity in production and comprehension

Three different types of ERPs were calculated and plotted: (1) the comprehension ERPs starting 1,100 ms before target word onset, (2) the production ERPs starting 1,100 ms before the go signal, and (3) the recalculated production ERPs starting 1,100 ms before speech onset. These were each analyzed by calculating the mean ERP amplitudes during the last 200 ms before the critical events (acoustic spoken word onset, go signal, and speech onset, respectively) for a selection of 30 frontoparietal electrodes (F7, F3, Fz, F4, F8, FT7, FC3, FCz, FC4, FT8, T7, C3, Cz, C4, T8, TP7, CP3, CPz, CP4, TP8, P7, P3, Pz, P4, P8, PO7, PO3, POz, PO4, PO8). To determine any differences between the two modalities (i.e., comprehension and production) and predictability (i.e., HC and LC), three four-way repeated measures ANOVA were performed. First, a repeated measures ANOVA was performed to compare the slow waves in comprehension and production aligned to beep sound onset [(1) vs (2), see above] using the following factors: modality (two levels: comprehension, production), predictability (two levels: HC, LC), gradient [six levels: anteroposterior, i.e., from frontal (F) to parieto-occipital (PO) regions], and laterality [five levels: left–right, i.e., from the most left (7) to the most right (8) electrodes]. Then the same 2 × 2 × 6 × 5 repeated measures ANOVA was computed on the same data extracted from the comprehension experiment (see above) and the slow waves aligned to speech onset in production [(1) vs (3), see above]. This last analysis was carried out both on the data with the AEPs and on the data corrected for the AEPs (see above, Ross et al., 2022).

Finally, a 2 × 2 × 2 × 6 × 5 repeated measures ANOVA with the additional factor semantic category (i.e., animals and tools) was computed to test whether the slow waves' topographies were modulated by the expected word category in both comhension and production. To this end, we focused this analysis on the slow waves preceding the perceived word in comprehension, and the speech onset in production as previous results have shown that the last time window (here the last 200 ms) before the critical event is when semantics modulations typically appear.

For all ANOVA results, we give *F*, degrees of freedom, and *p* values. In addition, we report an effect size measure (partial eta squares, *ηp*^2^; Cohen, 1973). For all significant effects involving more than two levels, we tested for sphericity violations, which, when detected, were corrected by means of the Greenhouse–Geisser (GG) correction (Greenhouse and Geisser, 1959). If corrections were made, GG epsilon values and corrected *p* values were reported.

##### Source estimation

The three models at test assume either comparable or different prediction-related cortical activation topographies in production and comprehension and for different semantic word types. To address these hypotheses, we restricted source estimations to the difference between the ERPs obtained in the predictable, HC condition and those in the unpredictable, LC condition. These prediction-related sources were compared between production and comprehension modalities and between tool and animal semantic categories. Distributed sources were estimated following the standard procedure in SPM 12 (Litvak and Friston, 2008). This method, as any other distributed source localization technique, cannot overcome the nonuniqueness of the inverse problem (Helmholtz, 1853), although it uses established priors for providing plausible source solutions for cognitive experiments. The cortical mesh of 8,196 vertices was obtained from the template MRI included in SPM, and it was coregistered with the electrode cap using three electrodes as fiducials: Fpz, TP9, and TP10; for the forward model, the “EEG BEM” was selected as the EEG head model. The four conditions [i.e., PP in comprehension before animal and tool nouns; PP in production before animal and tool nouns] were inverted together at the group level, using the “greedy search” algorithm (Bayesian approach; Friston et al., 2008; Litvak and Friston, 2008). The unexplained variance of the solutions reported in this article was below 10% (average ∼ 7.8%), which represents a realistic estimate in line with previous reports (Miozzo et al., 2015). The activations maps were smoothed using the Gaussian kernel of FWHM of 20 mm and then submitted to paired *t* tests to evaluate the critical comparisons.

We performed paired *t* tests to compare the brain responses elicited by the two modalities (comprehension vs production, collapsed across semantic categories) and by the two predictable semantic categories (animal vs tool words, collapsed across modalities). For all these comparisons, we compared the clusters of activations by considering both the whole-brain (*p* < 0.001, uncorrected) and hypothesis-driven regions of interest (ROIs). ROI analyses were carried out by applying a mask image, created with WFU_PickAtlas (Maldjian et al., 2003), which included the posterior, visual, and prefrontal motor brain areas (Brodmann areas 1, 2, 3, 4, 6 17, 18, and 19). This mask image was used as an explicit mask in the paired *t* tests to restrict the number of comparisons to these relevant voxels; for all these restricted contrasts, the *p* values were thresholded at *p* < 0.05 (FWE corrected; Table 1).

**Table 1.** Results: source analysis

	*x*	*y*	*z*	*t*-values (peak level)	Number of voxels	Brodmann areas	Cortical areas
PP sources computed on the difference signal HC minus LC: Comprehension > production (word type collapsed) Whole-brain analysis and ROIs (Brodmann areas 1, 2, 3, 4, 6, 17, 18, and 19) *p* < 0.001 uncorrected	-	-	-	n.s.	n.s.	-	-
PP sources computed on the difference signal HC minus LC: Production > comprehension (word type collapsed) Whole-brain analysis and ROIs (Brodmann areas: 1, 2, 3, 4, 6, 17, 18, and 19) *p* < 0.001 uncorrected	-	-	-	n.s.	n.s.	-	-
PP sources computed on the difference signal HC minus LC: animals > tools (modality collapsed) Whole-brain analysis *p* < 0.001 uncorrected	−20	−88	6	3.74	141	18	Posterior, visual cortex
PP sources computed on the difference signal HC minus LC: animals > tools (modality collapsed) ROIs (Brodmann areas: 1, 2, 3, 4, 6, 17, 18, and 19) *p* < 0.05 FWE corrected	−22	−90	4	3.75	48	18	Posterior, visual cortex
PP sources computed on the difference signal HC minus LC: tools > animals (modality collapsed) Whole-brain analysis *p* < 0.001 uncorrected	−40	−30	44	3.54	35	2/4	Somatosensory, motor cortex
PP sources computed on the difference signal HC minus LC: tools > animals (modality collapsed) ROIs (Brodmann areas: 1, 2, 3, 4, 6, 17, 18, and 19) *p* < 0.05 FWE corrected	−42	−30	42	3.69	64	2/4	Somatosensory, motor cortex

The table displays the significant clusters underlying the PPs recorded from the comprehension and production experiments. The Montreal Neurological Institute coordinates of the voxel with the highest *t* value, its *t* value, the number of significant voxels per each significant cluster, the Brodmann area labels, and a description of the area in which the cluster (peak voxel) was observed are reported.

##### Correlation analyses

Since previous results (Grisoni, 2022) showed the largest effect of predictability in left frontal areas, correlation analyses focused on this region and three more control areas. To this end, four ROIs were defined as the average of two neighbor electrodes (left frontal, F3, FC3; centrofrontal, Fz, FCz; left parietal, P3, PO3; centroparietal, Pz, POz). For these correlations, we focused on the time intervals before word presentation in the comprehension experiment and before speech onset in the production experiment obtained by removing the ICs related to the beep presentation.

To study whether the brain responses obtained reflect the predictability of the target word based on the sentence fragment, we correlated the predictive electrophysiological responses with both the reaction times (RTs) and the cloze probabilities (CPs). Response times can be considered an index of the ease with which participants were able to complete the sentence fragments; the context scores include information about the likelihood with which the target word appears in the sentence fragment context in ordinary language. Therefore, both can be used as measures of (possibly different aspects of) the predictability of the target word. To make it possible to obtain correlations across sentence fragments, averages per item were calculated across all participants; likewise, response times were averaged per item and across subjects. The correlations were also calculated between the ERPs obtained in the production and comprehension experiments.

Indeed, since the HC and LC sentences were matched for several features (see above), both the RTs and the contextual constraint scores can be considered a reliable measure of the facilitating effect that the preceding sentence fragments play on predictive processing, or, in other words, the more predictable a context, the faster the response and the higher the contextual constraint. Therefore, to test whether this predictive facilitation effect was functionally linked with our electrophysiological responses, we performed Pearson’s correlation analysis at item level between the RTs and the contextual constraint (e.g., average of the RTs of all the participants per each sentence) and the two anticipatory responses (i.e., average of the electrophysiological responses of all participants per each sentence fragment). Finally, we also performed Pearson’s correlation analysis between PPs and RPs at the item level.

Since each correlation was calculated for four ROIs, all the *p* values were multiplied by 4 to obtain the Bonferroni-corrected *p* values, which are those reported in the Results section. Finally, per each significant correlation, we also performed nonparametric Spearman rank order correlations to further test the relationships between our variables.

## Results

### Cloze probability and RTs in the speech production experiment

From the audio files recorded during the production experiment (see Materials and Methods), it was possible to extract the target words used by all participants; their cloze probability in the context of the sentence fragments was determined based on a previous study (Grisoni et al., 2021). RTs were obtained by measuring the time between the go signal and articulation onset. To reassess the cloze probability of our sentence fragments (Grisoni et al., 2021), we performed a 2 × 2 repeated measures ANOVA with the factors predictability (i.e., HC and LC) and semantic category (animals, tools), which revealed only a statistically significant difference between HC and LC conditions (main effect of predictability: *F*_1,28_ = 248.77, *p* < 0.001, *ηp*^2^ = 0.90; Fig. 1*c*). The most frequently uttered word in any of the HC fragments always corresponded to the word also presented in the comprehension experiment. The RTs were calculated as the time interval (Δ*t*) between the onset presentation of the beep (i.e., the “go” signal) and the speech onset of the pronounced word (Fig. 1*b*). These RTs were taken as an indirect index of sentence predictability, that is, as an index of the ease with which participants were able to complete each sentence fragment. Consistent with previous reports (Staub et al., 2015), the 2 × 2 (i.e., semantic category, predictability) repeated measures ANOVA revealed that participants were faster (on average, ∼245 ms) to complete the HC than the LC fragments (main effect of predictability: *F*_1,25_ = 33.62, *p* < 0.001, *ηp*^2^ = 0.57; Fig. 1*d*), thus confirming that predictability is associated with faster language processing (Huettig, 2015; Staub et al., 2015). For both the ANOVAs (i.e., cloze probability and RTs), no statistically significant difference involving the semantic category was found (i.e., neither the main effect of semantic category nor an interaction between semantic category and predictability), thus suggesting that the predictive constraints (HC, LC) were similarly manipulated across the two semantic categories (animal and tool nouns).

### ERP analysis

#### Anticipatory activity before the critical events

Electromyographic (EMG) activity showed no significant differences between HC and LC conditions during the last 200 ms before the critical events (in comprehension before final word onset: HC vs LC, *t*_25_ = −0.25, n.s.; in production before beep onset: HC vs LC, *t*_25_ = −0.7, n.s.; in production before speech onset: HC vs LC, *t*_25_ = −0.9, n.s.) nor between the two modalities (all *t*_25_ < 1.16, n.s.); this indicates that the amount of articulatory motor activity was similar across all the conditions.

**Figure 2.**
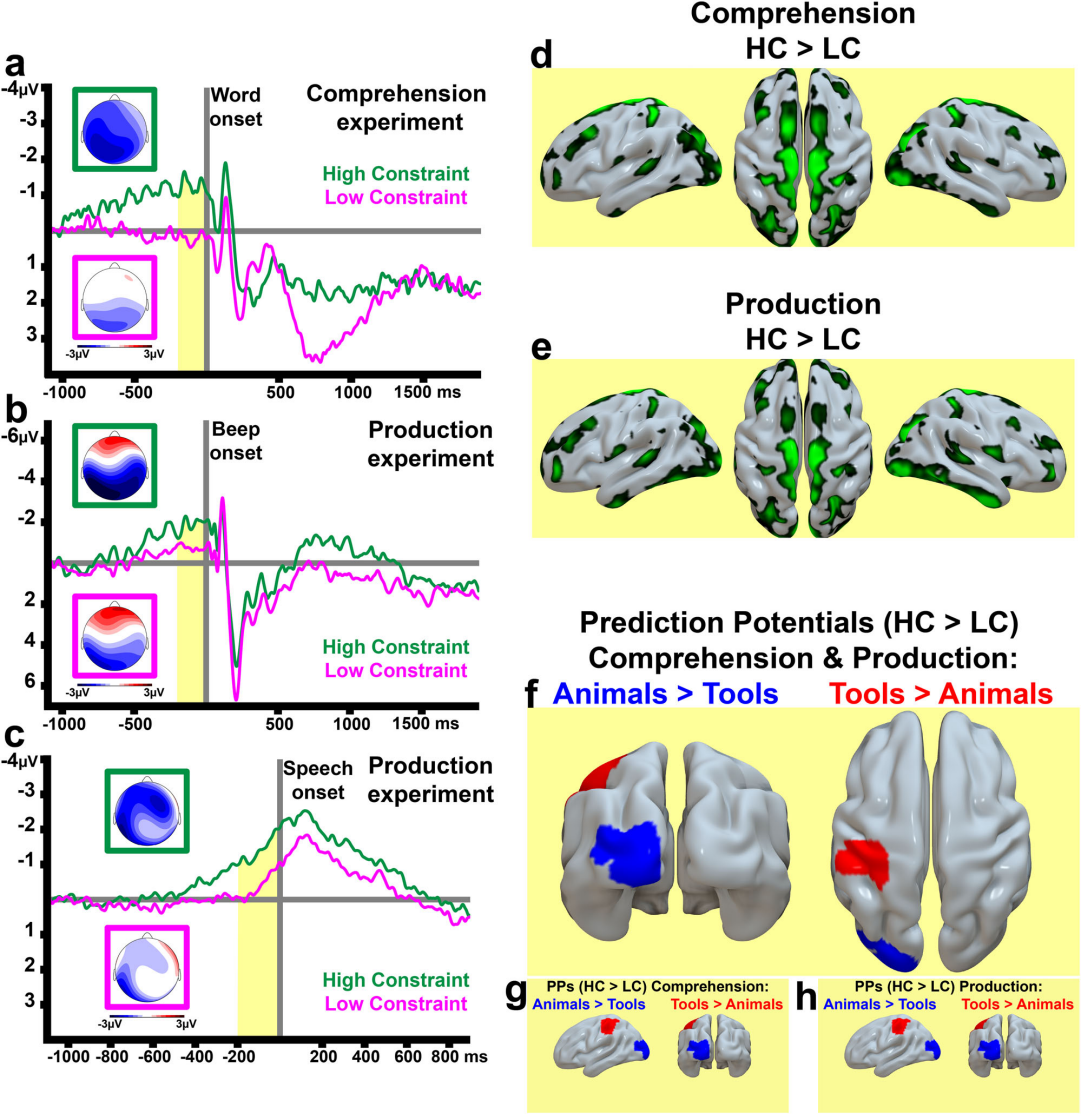
ERPs and source estimations. ***a***, PP observed at left frontal electrodes before the critical, final, word presentation of HC (green) and LC (magenta) sentences along with their voltage maps (average across the last 200 ms before critical word onset). ***b***, PP observed in the speech production experiment before the beep sound recorded at central electrodes; and (***c***) before speech onset at the left frontal region. All source estimations were computed on the difference ERPs (i.e., HC minus LC). Averages of source images from the comprehension experiment (***d***) and from the production experiment (***e***), the arbitrary scale is the same in the two modalities (***d***, ***e***). ***f***, Semantic contrasts (modality collapsed) animals > tools (blue) and tools > animals (red) left posterior view, right superior view. ***g***, ***h***, Differences (not thresholded) at ROIs [obtained from the significant results where the two modalities were collapsed) in the comprehension (***g***) and production (***h***) experiment for the two semantic contrasts (i.e., animals > tolls (blue); tools > animals (red)].

The repeated measures ANOVA on the mean amplitudes extracted from the comprehension experiment during the last 200 ms before critical word presentation and from the production experiment during the last 200 ms before beep sound onset showed that the slow waves were modulated by predictability (main effect of predictability: *F*_1,25_ = 6.06, *p* = 0.02, *ηp*^2^ = 0.20) and had different topographies across the scalp (main effects of gradient, that is anteroposterior: *F*_5,125_ = 23.72, GG epsilon = 0.33, adjusted *p* < 0.001, *ηp*^2^ = 0.49). In addition, the same ANOVA also revealed interactions of the factors modality (i.e., comprehension vs production), topography (i.e., gradient or laterality), and predictability (modality and gradient: *F*_5,125_ = 6.83, GG epsilon = 0.38, adjusted *p* = 0.003, *ηp*^2^ = 0.21; modality, predictability, and laterality: *F*_4,100_ = 2.88, GG epsilon = 0.63, adjusted *p* = 0.05, *ηp*^2^ = 0.10). Whereas the former interaction was due to larger anticipatory signals in comprehension than in production at the most frontal (i.e., *p* < 0.001 Bonferroni corrected) but neither at central nor at the most posterior locations, the latter indicated that the predictability effect (i.e., HC > LC) was more left-lateralized in comprehension than in production. Overall, these results showed a similar PP modulation for the two modalities (i.e., larger slow-wave responses for HC than LC contexts, Fig. 2*a,b*), with one notable difference: the topographical distribution of this modulation differed between the two modalities. However, as the go signal and beep likely give rise to additional sensory and cognitive processing (related to search, selection, response inhibition, etc.), the analysis of go signal–related activity was not strongly interpreted. Instead, we focus on the production results just before word articulation onset. Note that this corresponds directly to the evaluation of precritical-word activity in the comprehension experiment. The repeated measures ANOVA on the mean amplitudes extracted during the last 200 ms before word presentation in comprehension and before speech onset in production revealed larger anticipatory activity after HC than LC sentence fragments (main effect of predictability: *F*_1,25_ = 11.84, *p* = 0.002, *ηp*^2^ = 0.32). In addition, there was a significant interaction of predictability and laterality (*F*_4,100_ = 4.77, GG epsilon = 0.69, adjusted *p* = 0.005, *ηp*^2^ = 0.16). The modality factor interacted with gradient and laterality (*F*_20,500_ = 4.75, GG epsilon = 0.42, adjusted *p* < 0.001, *ηp*^2^ = 0.16), thus suggesting different ERP topographies in anticipation of comprehension and production. However, crucially, there was no significant interaction between predictability (factor predictability) and modality or a higher-order interaction involving both of these factors. Note that this would have been expected in the case of different prediction-related topographies. To further confirm the similarity of prediction-related ERP topographies between the production and comprehension modalities, we here also list the recording sites where significant differences between predictable and unpredictable sentences (HC vs LC) were obtained. When calculating prespeech onset slow waves, such differences appeared at recording sites (F7, F3, Fz, F4, F8, FT7, FC3, FCz, FC4, C3, Cz, C4, CP4). In comparison, such differences appeared in comprehension at recording sites (F7, F3, Fz, F4, FT7, FC3, FCz, FC4, T7, C3, CP3). As the critical difference (i.e., HC > LC) emerged at similar (frontal and central) locations in the two modalities, these results suggest that similar generators underly predictive processing in production and comprehension (Fig. 2*a,c*). The repeated measures ANOVA on the mean amplitudes extracted during the last 200 ms before word presentation in comprehension and speech onset in production (adjusted for the AEPs) revealed a further main effect of the factor gradient (*F*_5,125_ = 4.39, GG epsilon = 0.32, adjusted *p* = 0.03, *ηp*^2^ = 0.15) and the interactions of the factors predictability and gradient (*F*_5,125_ = 3.53, GG epsilon = 0.30, adjusted *p* = 0.05, *ηp*^2^ = 0.12) and gradient and laterality (*F*_20,500_ = 2.74, GG epsilon = 0.37, adjusted *p* = 0.009, *ηp*^2^ = 0.10). Finally, the same ANOVA performed on the mean amplitudes extracted during the last 200 ms before word onset in comprehension and speech onset in production not adjusted for the AEPs revealed similar results. This repeated measures ANOVA showed the main effects of predictability (*F*_1,25_ = 13.57, *p* = 0.001, *ηp*^2^ = 0.35) and gradient (*F*_5,125_ = 3.61, GG epsilon = 0.34, adjusted *p* = 0.04, *ηp*^2^ = 0.13) and the following interactions: predictability and gradient (*F*_5,125_ = 5.32, GG epsilon = 0.29, adjusted *p* = 0.02, *ηp*^2^ = 0.18), predictability and laterality (*F*_4,100_ = 4.59, GG epsilon = 0.69, adjusted *p* = 0.007, *ηp*^2^ = 0.16), gradient and laterality (*F*_20,500_ = 2.96, GG epsilon = 0.37, adjusted *p* = 0.005, *ηp*^2^ = 0.11), modality, gradient, and laterality (*F*_20,500_ = 5.29, GG epsilon = 0.42, adjusted *p* < 0.001, *ηp*^2^ = 0.17).

#### Predictive semantic processing in language comprehension and production

We also tested whether the semantic category of the predicted word affected the scalp distribution of both signals. We observed a significant interaction of the factors semantic category (i.e., animals and tools) and gradient (anteroposterior; *F*_5,125_ = 5.29, GG epsilon = 0.48, adjusted *p* = 0.005, *ηp*^2^ = 0.17), but not with modality. This interaction suggests general ERP-distributional differences between (HC and LC) conditions along the anteroposterior axis between animal and tool word conditions. Most importantly, however, there was a further significant triple interaction involving the factors semantic category, gradient, and predictability (*F*_5,125_ = 3.99, GG epsilon = 0.37, adjusted *p* = 0.03, *ηp*^2^ = 0.14), which provides strong evidence that the prediction-related difference between HC and LC stimuli was manifest in different ERP distributions for animal- and tool-related stimuli. The lack of significant interactions involving the two factors predictability and modality, or the triplet predictability, semantic category, and modality, is consistent with the assumption that similar prediction-related semantic activations occurred in both modalities.

The same sentence fragments were presented in the production and comprehension experiments. Therefore, stimulus repetition may have affected brain activity in production and comprehension, either in the same way or to different degrees. To explore any general or condition-specific repetition and possibly learning effects, we performed additional analyses. To this end, the additional ANOVAs were performed with the abovementioned factors plus an additional between-group variable experiment order (i.e., participants who started with the comprehension experiment vs participants who started with the production experiment). This new variable did not produce significant main effects in any of the analyses nor was it involved in significant interactions with other factors. In particular, there was no evidence of a differential effect of repetition on production and comprehension. These results fail to support a general influence of stimulus repetition on the PP in the present experiment or a differential influence on the ERPs obtained for the modalities or semantic categories. Our data do not support a specific influence of stimulus repetition or learning.

### Source estimation

Source estimations were computed on the slow waves obtained by subtracting the ERPs induced by LC fragments from those induced by the HC stimuli. Here, neither the comprehension > production nor the production > comprehension contrasts revealed significant clusters of activation (neither on the whole-brain nor in posterior, visual, and prefrontal ROIs, Fig. 2*d,e*, see also Materials and Methods). In contrast, the semantic difference (animal vs tool word condition, collapsed across modalities) revealed significant clusters in modality preferential brain areas. The contrast animals > tools revealed clusters of activations in posterior visual, regions in the left hemisphere; whereas the opposite, tools > animals, contrast showed greater activity in central sensorimotor areas (both exploratory whole-brain analysis *p* < 0.001 uncorrected and within ROIs *p* < 0.05 FWE corrected, see below Materials and Methods, Table 1, Fig. 2*f*). Strikingly similar prediction-related category-specific semantic differences in activation were seen in these ROIs for the two modalities (Fig. 2*g,f*). These results from ERP and source analyses are consistent with the assumption of little or no neurophysiological differences between prediction mechanisms effective in production and perception and with the participation of wide cortical areas and distributed semantic circuits in the prediction process.

### Correlation analysis

**Figure 3.**
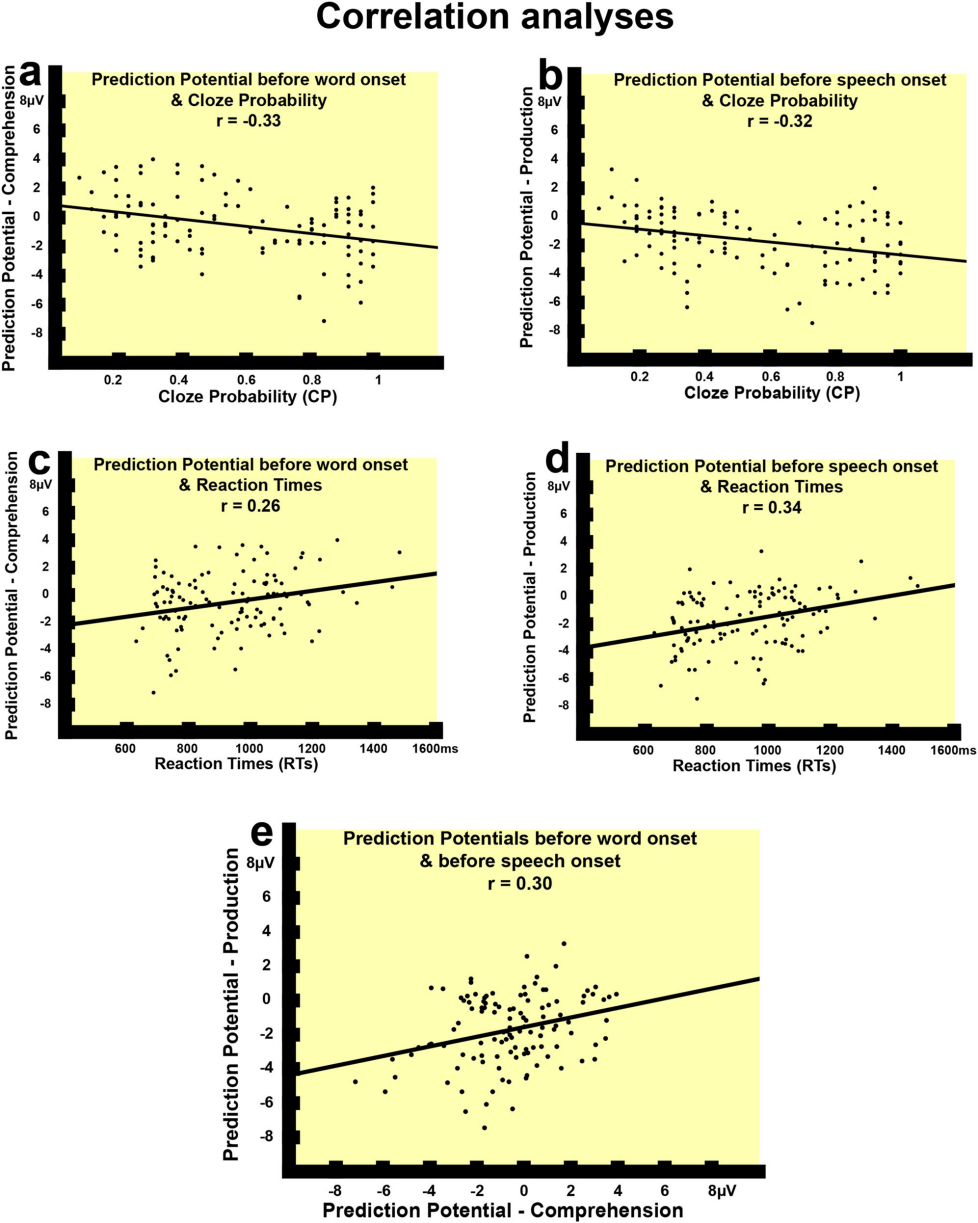
Results of correlation analyses. ***a***, ***b***, Significant correlations between the PP mean amplitudes recorded at the left frontal electrodes during the comprehension experiment, and the CP (***a***) and RTs (***c***) obtained from the speech production experiment; (***b***, ***d***) significant correlations between the PP mean amplitudes (aligned to speech onset) recorded at left frontal electrodes at sentence level in the speech production experiment and the CP (***b***) and the RTs (***d***) were obtained from the same experiment; (***e***) significant positive correlation between the two anticipatory signals from the two modalities recorded at sentence level at left frontal electrodes.

RTs and CPs extracted from the production experiment (see above and Materials and Methods) were used as a measure of the ease with which each sentence was completed by the participants; these measures were then considered as a measure of sentence fragment predictability, that is, the more predictable a fragment was, the higher the CP and the faster the participants were expected to complete the fragment after the beep (low RTs), “go,” sound (Fig. 1*d*). First, correlation analyses were performed to investigate whether the predictive signals were associated to these behavioral indexes of predictability. At sentence level (i.e., by item) both the slow waves in comprehension and production (before speech onset) correlated with these measures of predictability (i.e., both CPs and RTs) at the same left frontal region. The CPs and the ERP amplitudes showed negative correlations with each other, i.e., the higher the contextual constraint was, the larger (i.e., more negative) was the activity indexes elicited by the sentence fragments in the two modalities (in comprehension: left frontal, *r* = −0.33, Bonferroni adjusted *p* < 0.001; Fig. 3*a*; in production: both left frontal, *r* = −0.32, Bonferroni adjusted *p* < 0.001, and centrofrontal, *r* = −0.24, Bonferroni adjusted *p* = 0.032 regions; Fig. 3*b*). These relationships were confirmed also by nonparametric, Spearman, correlations (in comprehension: left frontal, *R* = −0.28, *p* = 0.002; in production: left frontal, *R* = −0.34, *p* < 0.001, and centrofrontal, *R* = −0.23, *p* = 0.01). In the same way, the RTs and the ERPs showed significant positive correlations (shorter RTs, more negative-going slow potentials). Specifically, the faster the participants completed a sentence after the “go” sound, the larger the predictive brain responses elicited by the sentence fragment in comprehension at left frontal electrodes (*r* = 0.26, Bonferroni adjusted *p* = 0.016, Fig. 3*c*) and in production at the same locations (*r* = 0.34, Bonferroni adjusted *p* < 0.001, Fig. 3*d*) and also at the frontocentral (*r* = 0.31, Bonferroni adjusted *p* = 0.004) ones. Nonparametric, Spearman, correlations confirmed these results (in comprehension: left frontal, *R* = 0.20, *p* = 0.03; in production: left frontal, *R* = 0.33, *p* < 0.001, centrofrontal: *R* = 0.27, *p* = 0.004). Overall, this analysis confirmed that the slow waves from the two modalities were associated with the ease with which each sentence fragment was completed. Furthermore, if the previous correlations are mediated by the same latent variable (i.e., predictability of the target event), then the prediction-related ERPs recorded in production and comprehension should also correlate with each other. To this end, we correlated the anticipatory signals obtained before word onset in production and comprehension between the two modalities and observed once again a significant positive correlation at the left frontal region (*r* = 0.30, Bonferroni adjusted *p* = 0.004; nonparametric: *R* = 0.24, *p* = 0.008; Fig. 3*e*), that is, at the location where the largest effect of predictability was observed (see above and Fig. 3*a–d*) and previously reported (Grisoni et al., 2021).

## Discussion

Here, we investigated anticipatory brain responses emerging after sentence fragments but before the onset of sentence-final spoken words in comprehension and before subjects started to pronounce the final word to complete the same fragments by producing a word of their own choice. Predictable, HC, sentence fragments induced larger anticipatory slow waves, or Prediction Potentials (PPs), than unpredictable, LC, fragments in both modalities: before sentence-final word onset in comprehension and before the two critical events in production (i.e., both before the “go” signal and before speech onset). Crucially, the topographies of the PPs measured in speech comprehension and production resembled each other. Similar prediction indices in production and comprehension were further suggested by source analyses showing congruent cortical activation patterns underlying these ERPs (Fig. 2*d,e*). In contrast to such congruency, there were clear topographical and source-level differences between the predictive brain activation patterns after sentence fragments predicting animal and tool words. Animal items activated posterior, visual, areas relatively more strongly, whereas tool items led to relatively enhanced predictive activity in frontocentral areas, including sensorimotor cortex. These differences are consistent with the literature on category-specific brain processing of animal and tool words presented in unpredictable contexts (Martin et al., 1996; Kiefer, 2005; Moseley and Pulvermüller, 2014; Kuhnke et al., 2020; Grisoni et al., 2021). Remarkably, these semantic differences between the prediction indices emerged in the same way in production and understanding (Fig. 2). In other words, the specific brain correlates of semantically specific predictions were similarly present across modalities.

These congruent patterns of brain responses suggest shared predictive processes across modalities. This is, of course, not to argue that there are no differences between producing a word form and understanding it when it is spoken by somebody else or, more generally, between production and perception. Clearly, the auditory system is stimulated in auditory perception, whereas the motor system is active in articulatory production, typically followed by auditory and tactile stimulation. These trivial differences must leave traces in brain activity, and the significant effects reported above for the factor modality are consistent with this. However, when we focussed on the specific brain index of prediction, the PP calculated by subtracting the ERPs before a matched unpredictable event from those of a predictable one (i.e., HC minus LC), there were no significant differences between the modalities.

It is evident that these data alone cannot provide proof of the null hypothesis, but considering the correlation analyses (Fig. 3) and the models of predictions at test (Introduction), there is no evidence in favor of the different-systems proposal (1) according to which the two modalities are controlled by different sets of cortical areas and, therefore, different cortical origins would be expectable in predictive language production and comprehension (Lichteim, 1885; Hickok and Poeppel, 2007; Price, 2012; Hickok, 2013). In contrast, we obtained strong support for the whole-brain, distributed-circuit, model (3) according to which predictions in both production and comprehension are mechanistically implemented in the same way by distributed neuronal assemblies, whose cortical topographies however may differ to reflect different types of stored semantic information. As strongly object-related animal nouns and action-related tool nouns draw relatively more strongly on visual versus action-related semantic information, respectively, the different distributions of the related circuits can be explained (Strijkers et al., 2017; De Lange et al., 2018; Grisoni et al., 2019b; Pulvermüller and Grisoni, 2020; Grisoni, 2022). The remaining model (2), which assumes that the same prediction area, or set of areas, is involved in any type of predictive processing (e.g., the sensorimotor system, Schubotz, 2007; Pickering and Clark, 2014), does not receive support from the present data as comparable prediction-related activations would have been forecasted not only in production and perception but likewise for different types of predicted information.

### Pre-articulation versus pre-go signal activity

Topographical differences between prediction-related ERPs emerged only when the production ERP was calculated relative to the go signal onset, where subjects may prepare for uttering the target word but also withhold it until the beep appears. This discrepancy between the two modalities might be explained by motor response inhibition (Diamond, 2013), which crucially depends on prefrontal areas (Aron et al., 2004, 2015; Wessel and Aron, 2017). Furthermore, our task left our participants the freedom to search for the to-be-uttered word already before the beep or spontaneously select an item after. As the task required all subjects to utter a word as fast as possible after the go signal, a task that they performed well, the appropriate time slot to check for preparatory and anticipatory processes was shortly before spoken word onset. Furthermore, the brain responses preceding the “go” signal might reflect the time point at which the target word has been selected, so that the earlier slow-wave onset in the predictable condition (HC) might just indicate an earlier onset of articulatory motor preparation in the HC than in the LC condition (Fig. 1*d*). When calculating predictive activity relative to speech production onset (and after the “go” signal), scalp topographies of the difference between HC and LC fragments were similar and statistically indistinguishable across modalities. Also, unlike the results observed before the “go” sound, the observation of larger PPs in HC than LC contexts during the last 200 ms before speech onset argues against the possibility that this modulation is merely due to an earlier latency of motor preparation, as they are both time-locked to speech onset.

### The prediction potential as an index of semantic expectancy

That the PP observed in the two modalities is an index of the predictability of an upcoming word was further confirmed by a range of correlation analyses. Not only did the CPs with which the target words follow the sentence fragments correlate with the amplitude of the PP recorded in production and comprehension experiments, but we also found similar significant relationships between PPs and RTs obtained from experiment participants. Together with earlier work (Grisoni et al., 2021), this further bolsters the PP's function as a modality-general prediction indicator (Pulvermüller and Grisoni, 2020). Further evidence for this came from studies relating the PP to established prediction error measures, such as the MMN and the N400, which showed functional relationships between the prediction-related (PP) and these prediction error–related brain responses (Grisoni et al., 2019a, 2021). These results are consistent with separable and systematically interrelated ERP indexes of prediction and prediction error, a feature required by predictive coding theories (Walsh et al., 2020).

A limitation of the current experiment may be the artificial character of the production experiment implementing a sentence completion task. However, it should be noted that our data indicate that the context (here HC vs LC sentence fragments) determined whether the critical event (spoken word in comprehension, speech onset in production) is firmly predicted in advance or accessed and selected on the spur of the moment (as indicated also by the variability with which participants completed the sentence fragments). It is therefore desirable for future research to study the brain basis of context-related predictions in more naturalistic speech production settings (Boux et al., 2021).

The PPs observed in the current experiments can be related to established ERP components. However, a range of negative-going slow waves have previously been shown to signal motor preparation—in particular the readiness potential or RP (Deecke et al., 1969; Shibasaki and Hallett, 2006; Schurger et al., 2021)—or to indicate foreseeable perceptions linked to motor preparation—such as the contingent negative variation (CNV; Walter et al., 1964; Luck and Kappenman, 2012). The RP has even been claimed to also precede predictable visual perceptions (Kilner et al., 2004), and likewise, the CNV may index stimulus expectation. One might therefore suggest that the ERPs we recorded in the production and comprehension experiments also be labeled RP and/or CNV, as they may primarily reflect motor preparation and perceptual expectation. We are not opposed to such nomenclature but should mention that such labeling would mask the obvious parallelism of the function of these slow waves in production and perception and likewise miss their important role in carrying predictive semantic information. This latter point, that the PP includes information about the semantic meaning of words (and, as shown in other experiments, different perceptual features of linguistic and nonlinguistic stimuli), seems to contrast with the function normally attributed to the abovementioned ERPs (e.g., the RP's role as a motor preparation index). We therefore prefer the label “PP” to highlight the cognitive aspects of this response.

The observation that, in addition to distributed cortical representations, prefrontal areas generally take an important role in predictive processing (Fuster and Bressler, 2015; Grisoni et al., 2017, 2021; León-Cabrera et al., 2019; Pulvermüller and Grisoni, 2020; Grisoni, 2022) is consistent with the proposed framework. Indeed, it is well-known that these prefrontal regions include multimodal and association areas (Fuster, 2001; Fuster and Bressler, 2015), and the reason why the PFC becomes important for predictive and semantic processing lies in its connectivity structure (Pulvermüller et al., 2021). Many areas of the prefrontal cortex (PFC) are connector hubs on which modality preferential areas converge. During predictive activation of these circuits, activity therefore emerges in prefrontal (and other) hub areas (Garagnani and Pulvermuller, 2013). This position can explain why predictive semantic processes generally draw heavily on the PFC (Grisoni, 2022). It is a valuable target of future research to work toward a mechanistic explanation of predictive processes, including the current neurophysiological indexes shared across production and comprehension.

## Conclusions

We found similar brain indexes of prediction in language production and comprehension, but consistently different distributed cortical sources when words with different meanings (animal and tool nouns) were predicted. These results are consistent with the proposal that the same distributed neuronal circuits are called into play when a word is predicted in production and understanding and therefore similar patterns of activation emerge in the two modalities (Pulvermüller and Fadiga, 2010; Strijkers et al., 2017). As the same differences between semantic category-specific predictive activity patterns were seen in production and comprehension, it appears unlikely that insufficient signal-to-noise ratios or lack of statistical power in the present study worked against finding between-modality differences. Our results confirm previous reports about reliable prediction-related brain responses which can be recorded similarly in both production and perception and may be generated by the preactivation of distributed neuronal circuits whose cortical topographies reflect semantic information immanent to the representations these circuits carry. The present results do not support models postulating different prediction mechanisms in production and perception or a narrowly localized prediction hub.

## Data Availability

We reported all data exclusions, all inclusion/exclusion criteria, all the preprocessing manipulations, and all the measures included in this work. The full list of stimuli used in this study (i.e., the sentence list) will be provided upon request, whereas behavioral and electrophysiological processed data used for the final analysis will be made available at Open Science Framework after publication.
